# A Novel Ligustrazine Derivative T-VA Prevents Neurotoxicity in Differentiated PC12 Cells and Protects the Brain against Ischemia Injury in MCAO Rats

**DOI:** 10.3390/ijms160921759

**Published:** 2015-09-09

**Authors:** Guoling Li, Yufei Tian, Yuzhong Zhang, Ying Hong, Yingzhi Hao, Chunxiao Chen, Penglong Wang, Haimin Lei

**Affiliations:** 1 School of Chinese Pharmacy, Beijing University of Chinese Medicine, Beijing 100102, China; E-Mails: Liguoliangsx@163.com (G.L.); tyf0323@126.com (Y.T.); limin516@163.com (Y.H.); cnhbhyz@hotmail.com (Y.H.); 2 Department of Pathology, Beijing University of Chinese Medicine, Beijing 100029, China; E-Mails: zyz100102@126.com (Y.Z.); springxiao-85@163.com (C.C.).

**Keywords:** ligustrazine derivative, neuroprotection, NF-κB/p65, VEGF, middle cerebral artery occlusion (MCAO)

## Abstract

Broad-spectrum drugs appear to be more promising for the treatment of acute ischemic stroke. In our previous work, a new ligustrazine derivative (3,5,6-trimethylpyrazin-2-yl) methyl 3-methoxy-4-[(3,5,6-trimethylpyrazin-2-yl)methoxy]benzoate (T-VA) showed neuroprotective effect on injured PC12 cells (EC_50_ = 4.249 µM). In the current study, we show that this beneficial effect was due to the modulation of nuclear transcription factor-κB/p65 (NF-κB/p65) and cyclooxygenase-2 (COX-2) expressions. We also show that T-VA exhibited neuroprotective effect in a rat model of ischemic stroke with concomitant improvement of motor functions. We propose that the protective effect observed *in vivo* is owing to increased vascular endothelial growth factor (VEGF) expression, decreased oxidative stress, and up-regulation of Ca^2+^–Mg^2+^ ATP enzyme activity. Altogether, our results warrant further studies on the utility of T-VA for the potential treatment of ischemic brain injuries, such as stroke.

## 1. Introduction

Stroke is the second most common cause of death and major cause of disability worldwide, particularly in the elderly [1]. Most strokes (80%) are ischemic and the disease relates with both cerebrovascular system and cranial nerves [2]. Ischemic stroke has exerted neurological disturbances, nerve damage and microcirculation damage in the brain, including oxidative stress, inflammatory response, and intracellular rise of Ca^2+^ [3,4].

In the light of recent knowledge, strategies such as antioxidants, calcium antagonists and angiogenesis appear to be effective in neuron damage, and therefore drugs with these categories will likely be more appropriate as adjunctive interventions for the treatment of acute ischemic stroke [5,6]. In recent years, it was suggested that some traditional Chinese medicine (TCM) and their products demonstrated protective effect against ischemic injury [7]. Thus, justifying TCM use in ischemic stroke patients is principally based on the synergic effect of their multi-categories [8,9].

**Figure 1:**
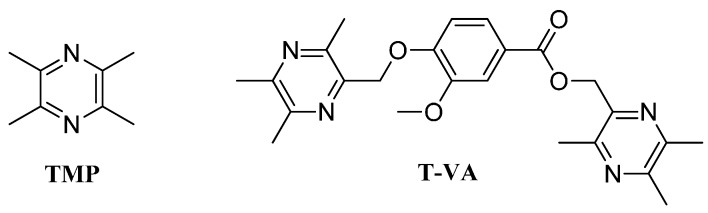
Chemical structures of TMP and T-VA.

The lack of effective and widely applicable pharmacological treatments for ischemic stroke patients may explain a growing interest in discovering drugs lead from traditional medicines [7]. Ligustrazine (2,3,5,6-tetramethylpyrazine) (TMP) (Figure 1), is one of the major effective ingredients of *Ligusticum chuanxiong* Hort., which is widely used in the treatment of ischemic stroke and cerebrovascular disease in Chinese communities for many years [10]. Previous studies have shown various pharmacological activities of TMP such as anti-inflammation, calcium antagonism, free radical-scavenging and antioxidant [11,12,13]. To further improve TMP’s neuroprotective properties, a series of novel ligustrazine-benzoic acid derivatives have been designed and synthesized using several neuroprotective ingredients from Chinese traditional medicinal herbs as starting materials. The results showed that ligustrazine-benzoic acid derivatives presented neuroprotective effects, on injured differentiated PC12 cells, of which, T-VA (C_24_H_28_N_4_O_4_) (Figure 1) displayed promising protective effect on the injured PC12 cells (EC_50_ = 4.249 μM) [14,15,16]. T-VA plasma concentration reached peak levels between 4 and 6 h post-administration times by intragastric method [17].

In the present investigation, we assessed the neuroprotective effect of T-VA on CoCl_2_-induced neurotoxicity in PC12 cells. Middle cerebral artery occlusion (MCAO) induced behavioral and neurological disturbances in rats were also evaluated. We further investigated the multi-categories underlying T-VA’s neuroprotective effects *in vitro*/*in vivo*.

## 2. Results

### 2.1. Cell Viability and (Hematoxylin-Eosin) HE Stains

**Figure 2:**
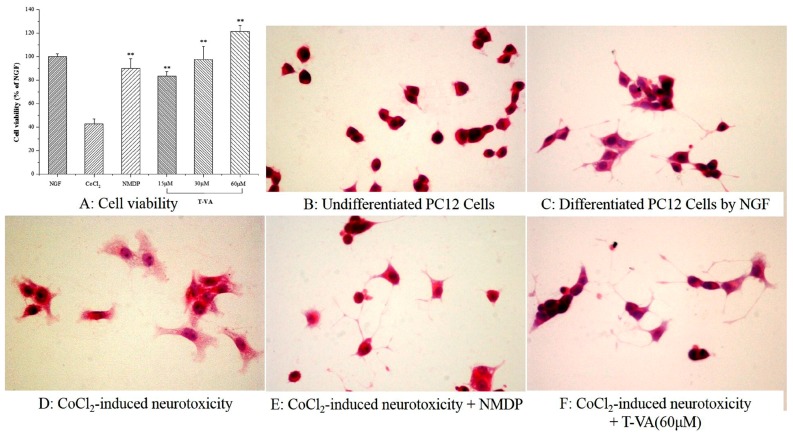
Effect of T-VA against CoCl_2_-induced damage in PC12 cells. (**A**) Survival of differentiated PC12 cells exposed to CoCl_2_; (**B**–**F**) effect of T-VA on the morphology of differentiated PC12 cells (×400); (**B**) control PC12 cells maintained under normal conditions; (**C**) PC12 cells differentiated by NGF; (**D**) differentiated PC12 cells exposed to CoCl_2_ insult; (**E**) differentiated PC12 cells pre-incubated with NMDP then exposed to CoCl_2_ insult; and (**F**) differentiated PC12 cells pre-incubated with T-VA (60 μM) then exposed to CoCl_2_ insult. Data are expressed as mean ± SD (*n* = 3). ** *p* < 0.01, compared with the model group.

Based on the fact that CoCl_2_ induces a hypoxic/ischemic condition, this study was designed to investigate the neuroprotective effect involved in CoCl_2_-induced cytotoxicity to PC12 cells [18]. Nimodiping (NMDP), used as strong calcium antagonist, has been previously shown to attenuate biochemical behavior and histopathological alterations from cerebral ischemia injury [19,20,21]. As shown in Figure 2A, T-VA attenuated CoCl_2_-induced damage in differentiated PC12 cells. With treatment of differentiated PC12 cells with CoCl_2_, the cell viability was reduced to 42.9% of the nerve growth factor (NGF) group value (100%), and NMDP increased model cell viability to 90.1%. The MTT assay revealed that T-VA significantly increased neurotoxic cell viability (83.3%, 97.6%, and 121.4% of the NGF group value, respectively) in a dose dependent manner, indicating that T-VA conferred protection against CoCl_2_-induced cytotoxicity to PC12 cells. As shown in Figure 2B, PC12 cells showed round cell bodies with intact and clear cell edges. By exposure to NGF, normal differentiated PC12 cells represented fine dendritic networks similar to those nerve cells (Figure 2C); In contrast, CoCl_2_-insulted differentiated PC12 cells developed mild cell body swelling and some cells shrunk the dendritic networks, and even lost neurites demonstrating round shape (Figure 2D). Pretreatment with NMDP (Figure 2E) and T-VA (Figure 2F) both alleviated morphological manifestations of cells damage and led to a pronounced increase in neurite-bearing cells compared to model cells.

### 2.2. The Expression of NF-κB/p65

**Figure 3:**
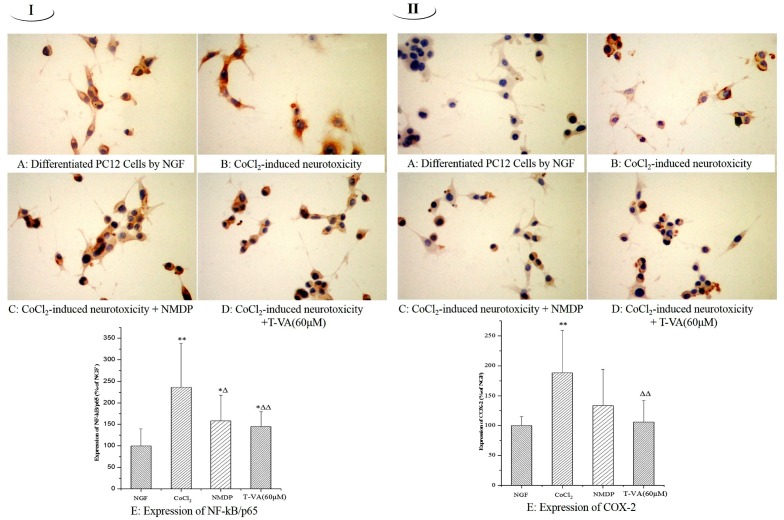
Effect of T-VA on the expression of NF-κB/p65 (**I**) and COX-2 (**II**) in PC12 cells (×400). (**A**) PC12 cells differentiated by NGF; (**B**) differentiated PC12 cells exposed to CoCl_2_ insult; (**C**) differentiated PC12 cells pre-incubated with NMDP then exposed to CoCl_2_ insult; (**D**) differentiated PC12 cells pre-incubated with T-VA (60 μM) then exposed to CoCl_2_ insult; (**I**-**E**) expression of NF-κB/p65 in PC12 cells; and (**II-E**) expression of COX-2 in PC12 cells. Data are expressed as mean ± SD (*n* = 3). * *p* < 0.05, compared with the NGF group; ** *p* < 0.01, compared with the NGF group; ^Δ^ *p* < 0.05, compared with the model group; ^ΔΔ^ *p* < 0.01, compared with the model group.

As shown in Figure 3-I, the expression of NF-κB/p65 (nuclear transcription factor-κB/p65) in model group (236.8%) was observably increased in the cytoplasm and cell membrane compared with the NGF group. However, pretreated with T-VA, the expression of NF-κB/p65 (144.7%) was decreased significantly compared with the model group, as well as NMDP (157.9%).

### 2.3. The Expression of COX-2

Through ICC staining, the positive COX-2 (cyclooxygenase-2) expression exhibited the fragmented nuclei in brown-yellow. As shown in Figure 3-II, model groups COX-2 expression (188.4%) was significantly increased by around twofold compared with the NGF group. In contrast, the expression of COX-2 was markedly decreased when PC12 cells were incubated with NMDP (133.3%). Meanwhile, the COX-2 expression was significantly attenuated by T-VA (105.8%) and similar to the NMDP group.

### 2.4. Acute Toxic Test in Vivo

**Figure 4:**
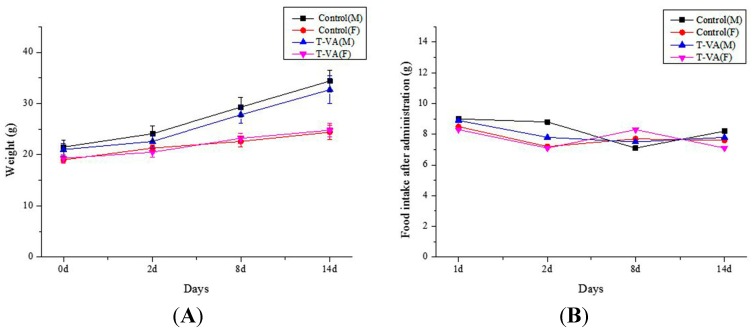
Physiological changes in mice during T-VA oral administration within two weeks. No significant differences were seen at time points between T-VA and control groups. (**A**) Body weights changes in mice during T-VA administration; (**B**) Food intake changes during T-VA administration. Data are expressed as mean ± SD (*n* = 10).

During T-VA oral administration of maximum tolerated dose (5.4 g/kg/day), all male and female mice showed no symptoms of toxicity or abnormal behavior within two weeks. At the end of the experiment, there were no significant differences in body weights and food intake between T-VA and control groups (Figure 4).

### 2.5. Behavioral Evaluation

#### 2.5.1. T-VA Reduced the Neurological Deficit Score

The repeated measures analysis (Table 1) revealed that MCAO rats showed a severe neurological deficit, whereas the sham-operated animals exhibited a normal reflex. NMDP group displayed an obvious reduction of the severity of this behavioral abnormality than the lesioned model. Moreover, neurological symptoms of T-VA groups were significantly alleviated, and T-VA (60 mg/kg) performance was comparable to that of NMDP group. Furthermore, it was demonstrated that TMP group also displayed some improvements in neurological deficit, although less pronounced than T-VA and NMDP.

#### 2.5.2. Beam Walking Test

The performance of beam walking test was evaluated on Day 3 and Day 7 after treatment (Table 1). Whereas no sensorimotor deficits were observed in the sham-operated rats, the MCAO animals showed significant deficits in motor performance on the beam-walking task in the session. When treated with NMDP, the motor impairment had significantly recovered, as compared to sham rats. Neurological symptoms of T-VA groups were significantly alleviated, and T-VA (60 mg/kg) performance was comparable to that of NMDP group. Moreover, it can be noted that T-VA groups exhibited better MCAO neurological motor recovery than TMP group in beam walking test.

#### 2.5.3. Bar-Grasping Test

In bar-grasping test, model rats demonstrated lesioned ability compared with sham group (Table 1). In contrast, T-VA groups showed significant neurological improvement compared to the model group on Day 3 and Day 7, as well as NMDP group. In addition, treated groups TMP also exhibited better function recovery, which was very similar to those of NMDP and T-VA groups. Furthermore, compares to TMP group, T-VA groups exhibited better motor function in bar-grasping evaluation.

**Table 1:** Effect of T-VA on the motor dysfunction in MCAO rats ($\bar{x}$ ± s).

Groups	Dose (mg/kg)	Neurologic Deficit Score		Beam-Walking Test Score		Grasping (g)	
		Ischemia for 3 Days	Ischemia for 7 Days	Ischemia for 3 Days	Ischemia for 7 Days	Ischemia for 3 Days	Ischemia for 7 Days
Sham	–	0 ± 0 **	0 ± 0 **	6 ± 0 **	6 ± 0 **	442.65 ± 62.84 **	531.44 ± 64.30 **
Model	–	2.14 ± 0.51	1.81 ± 0.56	1.47 ± 0.84	1.92 ± 2.02	317.01 ± 53.81	285.58 ± 80.02
NMDP	30	1.35 ± 0.39 *	1.11 ± 0.48 *	2.71 ± 1.49 **	3.50 ± 1.71 *	409.08 ± 65.13 **	393.08 ± 59.61 *
T-VA	60	1.46 ± 0.41	1.26 ± 0.35 *	3.6 ± 1.76 **	3.83 ± 1.70 *	395.50 ± 16.88 **	468.81 ± 78.14 **
T-VA	120	1.57 ± 0.37	1.34 ± 0.44	2.88 ± 1.62 **	3.20 ± 1.66	386.57 ± 42.35 **	421.68 ± 59.87 **
TMP	37	1.63 ± 0.4	1.45 ± 0.48	2.86 ± 1.79 *	2.40 ± 2.32	415.19 ± 26.81 **	434.22 ± 50.23 **

Data are expressed as mean ± SD (*n* = 10). * *p* < 0.05, compared with model group. ** *p* < 0.01, compared with model group.

### 2.6. Effect of T-VA on Histological Alterations

**Figure 5:**
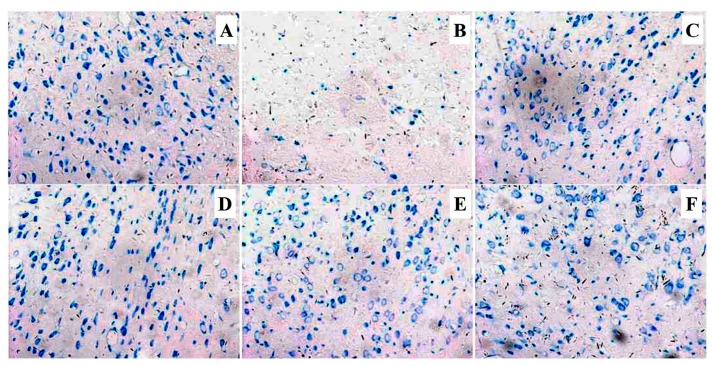
Representative microphotographs of nissl’s staining in the hippocampus of rat brain sections (×200): (**A**) Sham; (**B**) Model; (**C**) NMDP; (**D**) T-VA (60 mg/kg); (**E**) T-VA (120 mg/kg); and (**F**) TMP.

Under nissl’s stain (Figure 5), model group displayed less nissl bodies in the hippocampal region, which were colored light blue, than sham operated group (Figure 5A). As shown in Figure 5B, the ischemic-induced nerve cell destruction and dissolution (chromatolysis) caused either a diffuse sparse staining. In contrast, NMDP group (Figure 5C) exhibited plenty of nissl substance colored in dark blue. In addition, spatial distribution features of nissl bodies located in neurons of T-VA groups were elevated dramatically, while TMP (Figure 5F) showed more survival nerve cells and nissl bodies compared with the MCAO vehicle treated group. Furthermore, it showed that T-VA group had a larger number of nissl bodies colored in darker blue than TMP group, which was comparable to sham group.

### 2.7. Effect of T-VA on VEGF in MCAO Rats

**Figure 6:**
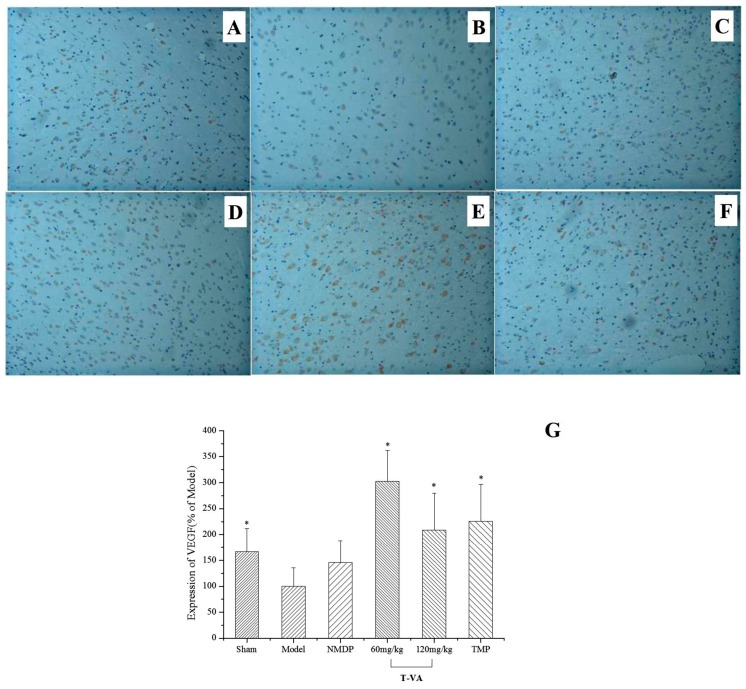
VEGF immunoreactivity of T-VA in MCAO rats. (**A**–**F**) Representative microphotographs of VEGF immunoreactivity in the hippocampus of rat brain sections (×200): (**A**) Sham; (**B**) Model; (**C**) NMDP; (**D**) T-VA (60 mg/kg); (**E**) T-VA (120 mg/kg); (**F**) TMP; and (**G**) effect of T-VA on IOD of VEGF determined by IHC in MCAO rats. Data are expressed as mean ± SD (*n* = 10). * *p* < 0.05, compared with the model group.

VEGF (vascular endothelial growth factor) expression was measured by integral optical density (IOD) determined by immunohistochemistry (IHC). Thirteen days post-MCAO, as shown in Figure 6, the level of VEGF decreased significantly in response to ischemic stroke. The NMDP (Figure 6C) group, which contained 145.8% VEGF intensity, exhibited a relatively weak immunoreactivity of VEGF. In addition, compared with the model group, VEGF immunoreactivity had noted up-regulation in TMP (Figure 6F, 226.0%). T-VA groups, VEGF immunoreactivity of the neurons was approximately to 302.5% of the model at 60 mg/kg (Figure 6D), and 209.1% at 120 mg/kg (Figure 6E). Moreover, it was shown that the protection induced by T-VA at 60 mg/kg was greater than that of other groups.

### 2.8. Detection of Superoxide Dismutase (SOD) Activity and Ca^2+^–Mg^2+^ATP Enzyme Activity of Ischemic Brain Tissue

As shown in Table 2, model rats elicited significant decreases of SOD activity levels, which substantially support the occurrence of oxidative damage in the pathogenesis of ischemic injury. However, compared with vehicle treated MCAO rats, T-VA and TMP significantly restored SOD activity, as well as NMDP.

As a positive feedback modulator of neuronal cell function by promoting Ca^2+^ migration in cerebral cortical neurons cells, Ca^2+^*–*Mg^2+^ATP enzyme expression was increased significantly compared with model rats. In contrast, NMDP dramatically enhanced Ca^2+^*–*Mg^2+^ATP enzyme activity, which was consistent with its calcium antagonist in clinic. Interestingly, T-VA and TMP groups also showed marked promotion, displaying satisfactory effect on Ca^2+^*–*Mg^2+^ATP enzyme activity.

**Table 2:** Effect of T-VA on SOD and Ca^2+^*–*Mg^2+^ATP enzyme activity in rats with MCAO ($\bar{x}$ ± s).

Groups	Dose (mg/kg)	SOD (U/mg Protein)	Ca^2+^–Mg^2+^ ATP Enzyme (μmolPi/(mg Protein/h))
Sham	–	61.85 ± 12.53 **	3.66 ± 1.29 *
Model	–	40.28 ± 7.51	5.32 ± 2.61
NMDP	30	79.85 ± 36.57 **	13.25 ± 5.73 **
T-VA	60	75.29 ± 30.08 **	8.91 ± 4.05 *
T-VA	120	85.03 ± 28.03 **	10.07 ± 5.15 *
TMP	37	61.58 ± 16.23 **	9.13 ± 4.11 *

Data are expressed as mean ± SD (*n* = 10). * *p* < 0.05, compared with model group; ** *p* < 0.01, compared with model group.

### 2.9. Statistical Analysis

All data are expressed as mean ± SD. Statistical comparisons between groups performed using one-way ANOVA followed by least significant difference (LSD) *post hoc* test and *p* < 0.05 was considered significant.

## 3. Discussion

NF-κB, ubiquitously distributed within the nervous system, is a transcription factor crucially involved in neuronal function [22]. Immunohistochemical staining showed that CoCl_2_ injury lead to an increase in COX-2 expression, as well as activating NF-κB [23]. Previous studies demonstrated that TMP exerted neuroprotection by reducing inflammatory cell activation and pro-inflammatory mediator production in the ischemic stroke model, which is consist with present results [24,25]. The results also showed that treatment with T-VA significantly reduced COX-2 protein expression and NF-κB activation. The mechanism of T-VA exerted its neuroprotection may include blockade of NF-κB activation and the subsequent suppression of COX-2 in the injured PC12 cells.

The safety of T-VA was tested to determine its potential as a drug lead [26]. In the present study, T-VA was found to be completely nontoxic to cultured PC12 cells up to a concentration of 60 μM. Furthermore, acute oral administration toxicity testing in mice showed no abnormal symptoms upon the maximum tolerated dose of T-VA (5.4 g/kg). Thus, T-VA is likely to be an orally safe agent.

Behavioral evaluation reflects dysfunction at multiple anatomical areas of nervous system. The neurological deficit score, beam-walking task and bar-grasping tests have the ability to assess fine-motor initiation, coordination and postural balance of an individual animal [27]. In the present study, MCAO rats demonstrated poor performances at all test sessions following the physical lesion, but treated groups showed a better functional outcome. It was especially notable that T-VA exhibit promising resistance to MCAO rats and improve motor function. Furthermore, T-VA had a more favorable effect than its parent compound TMP on behavior functional test. The reason for the beneficial effect of T-VA on function recovery was unclear, but several factors may be involved [28].

Morphological features have been reproducibly detected in the ischemic brain. In the current study, when treated with T-VA, histopathology showed significant population of neurons restored within the ischemic core areas, as evidenced by the nissl staining. Histopathological analyses were in agreement with previous study that TMP provide neuroprotection against ischemic brain injury through prevention of neuronal loss [11]. Thus, T-VA displayed the ability to attenuate neurons loss for the treatment of ischemic brain injury.

VEGF, a major mediator of angiogenesis, is an important stroke-related pathogenic factor stimulating neurogenesis, promoting growth of neurons and protecting neuronal tissues in response to ischemia [29,30]. VEGF is toxic early but beneficial late post-stroke, which served as a useful positive control [31]. When treated with T-VA, VEGF up-regulated dramatically in the recovery period after ischemic stroke, thereby inducing endothelial proliferation, vascular density enlargement and neuron recovery, further preventing MCAO rats from ischemic injury.

Oxidative stress is proposed as a fundamental mechanism of brain damage in ischemia stroke [32]. Biological substances SOD, correlated with the size of ischemic stroke and clinical outcome, have been investigated as indirect markers of neuron damage [32,33]. In the present study, we found TMP restored the tissue levels of SOD, which was consistent with its role as an antioxidant [12,34]; T-VA was protective against oxidative stress damage via enhancing the activities of SOD after MCAO, suggesting a promising approach to limit the extent of damage of ischemia injury.

Stroke is related to a disturbed calcium homeostasis as a consequence of a deranged energy metabolism [4,35]. In terms of neural activity, it is well known that Mg^2+^, which plays an important role in biological functions, blocks the NMDA receptor, then attenuate Ca^2+^ overload [36,37]. There is accumulative evidence that Ca^2+^–Mg^2+^ATP enzyme play a pivotal role in pathophysiological progress of stroke [38]. The low expression of Ca^2+^–Mg^2+^ATP enzyme leads to intracellular Ca^2+^ and Mg^2^ imbalances, thereby further worsening the ischemia injury [35]. Following T-VA treatment, the immunoreactivity of Ca^2+^–Mg^2+^ATP enzyme was markedly increased, thereby attenuating intracellular Ca^2+^ overload. It indicated that T-VA could target the intracellular Ca^2+^ overload, which served as an important therapeutic strategy to repair damage of nerve cells, consequently improving cerebral energy metabolism and brain function [12,39,40].

It was previously reported that T-VA congener structures also exhibited potential antithrombosis [41]. Therefore, T-VA could be more effective in appropriately selected stroke patients via combinations of thrombolytic and neuroprotective therapies [42].

## 4. Experimental Section

### 4.1. General

The PC12 cell line was purchased from Institute of Materia of Chinese Academy of Medical Science. Cells and were cultured in RPMI 1640 medium supplemented with 5% (*v*/*v*) fetal bovine serum (FBS), 10% (*v*/*v*) heat inactivated horse serum and 100 U/mL penicillin-streptomycin (Thermo Technologies, Waltham, MA, USA) and incubated at 37 °C in a humidified atmosphere of 5% CO_2_. Female/male ICR mice and male SD rats (Beijing Vital River Laboratory Animal Technology Company Limited, Beijing, China) were kept under standard laboratory conditions (tap water, constant room temperature, 22 °C). All animal protocols were approved by the Ethics Committee of Beijing University of TCM in accordance with the National Institutes of Health guidelines (Contract 2012-0036, June 2012). All efforts were made to minimize animal suffering, and to reduce the number of animals used.

### 4.2. Cell Viability Assay and HE Staining

Cell viability was tested according to our previous studies. Briefly, PC12 cells, maintained with serum-free media for 14 h, were placed on 96-well dishes pre-coated with poly-l-lysine at 7 × 10^4^ cells/mL, differentiated by treatment with NGF (50 ng/mL). Thereafter, the differentiated PC12 cells were pretreated with NMDP (30 µM) and T-VA (60, 30, 15 µM) for 36 h, respectively; then it was exposed to CoCl_2_ for 12 h. After the treatments, MTT solution (20 μL, 5 mg/mL) was added to the culture medium and incubated at 37 °C for 4 h. The formazan crystal was solubilized in DMSO and the OD was read for optical density at 490 nm (Thermo Multiskan GO, Waltham, MA, USA). Cell viability was expressed as a percentage of the control.

HE stains was measured according to our previous study [14,43]. The cellular morphology and percentage of cells showing neurite outgrowth was determined by light microscopy (Nikon, Kobe, Japan).

### 4.3. The Expression of NF-κB/p65 and COX-2

Immunohistochemical study was performed with monoclonal antibodies against NF-κB (P65) and COX-2. The final dilution for these antibodies was 1:50 and 1:150, respectively. The slides of cells were prepared as HE staining and the expression of NF-κB/p65 and COX-2 was performed as per manufacturer’s instructions (Beijing Kangwei Century Biological Technology Ltd., Beijing, China). Cells were observed and photographed under a microscope.

### 4.4. Acute Toxicity

ICR mice of both sexes, weighing 18–22 g, were divided into two groups of 20 animals matched in weight and size. The maximum tolerated dose (45 mg/mL) of T-VA was prepared in 0.3% CMC-Na solution. Then, one group of 20 mice of male and female pre-deprived of food for 24 h were orally administered the maximum tolerated dose, 0.4 mL/10 g, 3 times during 12 h. Similarly, 20 mice in control group were given suspension as described above. The general behavior of the mice was observed continuously for 1, 4 and 24 h after the treatment. The mice were further observed up to 14 days. Behavior, toxic effects and mortality response were recorded.

### 4.5. Animal Models for Transient Focal Cerebral Ischemia

Transient MCAO was conducted in male SD rats (280–300 g) according to Koizumi and Zea [44,45]. In brief, male SD rats were anesthetized with 10% chloral hydrate (350 mg/kg). The right common carotid artery and external carotid arteries were isolated and ligated. A nylon monofilament (diameter 0.25 mm) was introduced from the common carotid artery into the internal carotid artery until a resistance was encountered (about 18 to 20 mm from the external–internal carotid artery bifurcation), thus blocking the middle cerebral artery and leading to cerebral ischemia. Then the muscle and skin were sutured with 4-0 nylon. Animals were given penicillin (4 million units/mL) during the three days after operation. The right common, internal, and external carotid arteries were exposed and separated in the sham group.

According to Zea L. score standard [45], the model was qualified when it scored 1–3 (score 0, normal, noneurologic deficit; score 1, failure to extend left forepaw fully; score 2, circling to the left; score 3, falling to the left; score 4, cannot walk spontaneously and had a depressed level of consciousness). Rats scored 0 and 4 were unqualified for the further test and abandoned.

Fifty animals that scored 1–3 were randomly assigned into five groups: model group; T-VA high-dose group (120 mg/kg once daily for 10 days); T-VA low-dose group (60 mg/kg once daily for 10 days); NMDP group (30 mg/kg once daily for 10 days); and TMP group (37 mg/kg once daily for 10 days). Another 10 rats that received all surgical procedures but without the suture inserted, were assigned to sham operation group. Three days after operation, sham group and model group were gavaged orally with 0.3% CMC-Na once daily for 10 days. The other groups were gavaged orally with different doses of drugs for 10 days.

### 4.6. Behavioral Evaluation

To assess sensory ability, motor performance, balance, and coordination of the MCAO rats, beam walking and bar-grasping tests were carried out as described below.

#### 4.6.1. Neurological Deficit Score

The neurological deficit score was evaluated as described according to Zea L. score standard (See above section: Animal models for transient focal cerebral ischemia). Then, mice were scored on Days 3 and 7 in the first week after MCAO.

#### 4.6.2. Beam Walking Test

The beam-walking apparatus consisted of a wooden beam (120 × 2 × 1 cm), elevated 100 cm above the floor. Rats were stimulated to walk on the beam to get into “goal box” fixed to the other side. Ability was measured using a modified scale: score 0, the rat is unable to stay on the beam; score 1, the rat is unable to move the body or any limb on the beam; score 2, the rat is unable to traverse the beam; score 3, the rat takes a considerable amount of time to traverse the beam because of difficulty walking with foot slip more than 50%; score 4, the rat shows disability of walking on the beam with foot slip less than 50%; score 5, the rat traverses with just one foot slip; and score 6, the rat traverses the beam with no foot slip. Before surgery, pre-training was carried out in which rats were initially handled and habituated to the testing room. Training was repeated five times (1 time/day) until all rats were skilled in the walking tasks. Measurements were taken on Days 3 and 7 after treatment.

#### 4.6.3. Bar-Grasping Test

Bar-grasping test was carried out by a tensile tester. Rats would grip the crossbar until they slipped, and the tension value was recorded. Each measure was repeated 3 times. Measurements were taken on Days 3 and 7 after treatment.

### 4.7. Pharmacological Analysis: Brain Tissue Preparation for Nissl Stains

The MCAO and sham operated rats were anesthetized with 10% chloral hydrate (0.35 mL/100 g, intraperitoneal injection), then penetrated with a perfusion cannula through the left ventricle. The rats were perfused with ice-cold saline for 15 min, followed by fixation with 4% paraformaldehyde (in 1× PBS) for 15 min. The brains were quickly removed from the skulls and stored in 4% paraformaldehyde (in 1× PBS). Before being embedded in paraffin, the brains were dehydrated through gradient ethanol and cleared in xylene. Brains were cut into 6 μm sections and stained with nissl. The sections were examined under a light microscope.

### 4.8. VEGF Expression in Rats with MCAO

Samples of ischemic brain tissue were obtained and treated according to the same procedure as above. Immunohistochemistry was performed as previously described [46]. In brief, the slices were dewaxed and washed three times with PBS. Then, the slices were immersed in heat induced antigen retrieval solution (0.1 M citric acid buffer solution) and cooled at room temperature. After three PBS washes, the slices were treated with 3% H_2_O_2_-CH_3_OH for 10 min to block endogenous peroxidase activity. After three PBS washes, sequential slides were incubated with the primary antibody (rabbit polyclonal anti-rat VEGF, 1:50) for approximately 2 h at 37 °C (PBS was used as a negative control). After three PBS washes, the slices incubated with the biotinylated goat anti-rabbit immunoglobulin secondary antibody for 50 min at 37 °C. Next, the slides were washed three times with PBS and developed with DAB kit (1:50, Zhongshan Golden Bridge Biological Technology Ltd., Beijing, China). Then, the slides were rinsed in distilled water to terminate the color. Finally, the slides were stained with hematoxylin and dehydrated in a graded alcohol series, followed by the addition of xylene and neutral gum cementing. The expression of VEGF was observed and photographed under a light microscope.

### 4.9. Detection of SOD Activity and Ca^2+^–Mg^2+^ATP Enzyme Activity of Ischemic Brain Tissue

After treatment for 10 days, animals were anesthetized with 10% chloral hydrate (350 mg/kg), and the ischemic hemisphere was dissected and homogenized with a physiological saline. The homogenates were centrifuged for 15 min at 3000× *g*, and the supernatant was used for measurement. The SOD activity was determined by a xanthine oxidase method. The procedures to quantify SOD and Ca^2+^–Mg^2+^ATP activity were carried out according to the description of the assay kits (Jiancheng Institute of Biologic Engineering, Nanjing, China).

## 5. Conclusions

In the present study, we demonstrated that T-VA protected against CoCl_2_-induced differentiated PC12 cell damage *in vitro*. The results showed that modulation of NF-κB and COX-2 in damaged PC12 cells were involved in the neuroprotective mechanisms of T-VA. In addition, T-VA exhibited potential resistance to MCAO rats and improved behavioral function. Moreover, the beneficial effect of T-VA on function recovery was possibly via increasing VEGF expression, inhibiting oxidative stress and up-regulating Ca^2+^–Mg^2+^ATP enzyme activity. All together, the results clearly indicate that T-VA is a potential candidate with multi-categories for intervention in ischemic brain injuries such as stroke.
