# Correction: Li, G., *et al*. A Novel Ligustrazine Derivative T-VA Prevents Neurotoxicity in Differentiated PC12 Cells and Protects the Brain against Ischemia Injury in MCAO Rats. *Int. J. Mol. Sci.* 2015, *16*, 21759–21774

**DOI:** 10.3390/ijms17040468

**Published:** 2016-03-29

**Authors:** Guoliang Li, Yufei Tian, Yuzhong Zhang, Ying Hong, Yingzhi Hao, Chunxiao Chen, Penglong Wang, Haimin Lei

**Affiliations:** 1 School of Chinese Pharmacy, Beijing University of Chinese Medicine, Beijing 100102, China; Liguoliangsx@163.com (G.L.); tyf0323@126.com (Y.T.); limin516@163.com (Y.H.); cnhbhyz@hotmail.com (Y.H.); 2 Department of Pathology, Beijing University of Chinese Medicine, Beijing 100029, China; zyz100102@126.com (Y.Z.); springxiao-85@163.com (C.C.)

The authors wish to change the first author’s name “Guoling Li” to “Guoliang Li” on the Page of their paper published in the *International Journal of Molecular Sciences* [1]. The authors apologize for any inconvenience. The manuscript will be updated and the original will remain online at the article webpage.
